# Evaluation of the *MC3R* gene pertaining to body weight and height regulation and puberty development

**DOI:** 10.1038/s41598-023-37344-1

**Published:** 2023-06-27

**Authors:** Yiran Zheng, Luisa Sophie Rajcsanyi, Triinu Peters, Astrid Dempfle, Stefan A. Wudy, Johannes Hebebrand, Anke Hinney

**Affiliations:** 1 Department of Child and Adolescent Psychiatry, Psychosomatics and Psychotherapy, University Hospital Essen, University of Duisburg-Essen, Virchowstr. 174, 45147 Essen, Germany; 2 Center for Translational Neuro- and Behavioral Sciences, University Hospital Essen, University of Duisburg-Essen, Essen, Germany; 3 Institute of Medical Informatics and Statistics, Kiel University, Kiel, Germany; 4 Division of Pediatric Endocrinology and Diabetology, Center of Child and Adolescent Medicine, Justus Liebig University, Giessen, Germany

**Keywords:** Obesity, Growth disorders, Obesity, Genetics

## Abstract

Recent studies reported an impact of the melanocortin 3 receptor (MC3R) on the regulation of body weight, linear growth and puberty timing. Previously, allele p.44Ile of a frequent non-synonymous variant (NSV) p.Val44Ile was reported to be associated with decreased lean body mass (LBM) and later puberty in both sexes. We Sanger sequenced the coding region of *MC3R* in 185 children or adolescents with short normal stature (SNS) or 258 individuals with severe obesity, and 192 healthy-lean individuals. Eleven variants (six NSVs) were identified. *In-silico* analyses ensued. Three rare loss-of-function (LoF) variants (p.Phe45Ser, p.Arg220Ser and p.Ile298Ser) were only found in severely obese individuals. One novel highly conserved NSV (p.Ala214Val), predicted to increase protein stability, was detected in a single lean female. In the individuals with SNS, we observed deviation from Hardy–Weinberg Equilibrium (HWE) (*p* = 0.012) for p.Val44Ile (MAF = 11.62%). Homozygous p.44Ile carriers with SNS had an increased BMI, but this effect did not remain significant after Bonferroni correction. In line with previous findings, the detected LoF NSVs may suggest that dysfunction in MC3R is associated with decreased body height, obesity and delayed puberty.

## Introduction

Maintaining energy homeostasis is essential for the survival of metazoans, which is achieved through the balance of energy intake and expenditure. Puberty is a short period but critical period of growth characterized by altered energy requirements and metabolism^1^. Energy balance is associated with growth velocity, skeletal maturation, and puberty timing^2,3^. Short normal stature (SNS) is defined as body height for age and sex (BH-centile) below the 5th percentile that is not due to readily detectable pathogenic reasons, such as chronic diseases, hormonal deficiencies, or dysmorphic syndromes^4^. Obesity in adults is defined by a body mass index (BMI, kg/m^2^) ≥ 30 kg/m^2^^5,6^ and in children or adolescents by BMI ≥ the 95th percentile for age and sex^7^. Wudy et al. showed that children and adolescents with SNS had a lower BMI and enjoyed food less than children without SNS^8^. Numerous studies have indicated that obesity is relevant for puberty timing^9,10^, and many genes that have been studied for stature are also relevant to the puberty timing^11,12^. One previous study reported frequent occurrences of delayed maturation in children with SNS^12^. Although there is no direct correlation between BMI and final body height^13,14^, some studies reported a positive correlation between adult obesity and short stature and reversed association in children and adolescents^15,16^.

The hypothalamic leptinergic melanocortinergic system is involved in energy metabolism^13,17,18^. The ,melanocortin 3 receptor (MC3R), which is a typical member of G protein-coupled receptors (GPCRs) superfamily and contains seven transmembrane helixes, is involved in this system^19^. Activation of MC3R by agonists lead to increased production of cAMP^19^, which has been extensively studied in central and peripheral regulation of energy homeostasis and nutrient partitioning^20^. Several loss-of-function variants (LoFs) in the MC3R gene (*MC3R*) have been found to decrease the activity of cAMP^21^. Mc4r and/or Mc3r knockout mice display an altered body weight regulation. Mice lacking the Mc4r are obese and have lower energy expenditure than wildtype mice^22,23^. In contrast, Mc3r deficient mice are not hyperphagic (normal food intake and metabolism levels), but they have increased fat mass (FM) and decreased lean body mass (LBM)^24,25^. Mc3r and Mc4r double knockout mice provide a deeper insight into the interaction between MCRs. Mc3r deficiency appears to exert an additive effect on Mc4r deficiency in several aspects, including higher lipid profile levels and severe glucose intolerance^26^. The distinct functions of MC3R and MC4R indicate a non-redundant metabolic pattern between them^27^.

The hypothalamic–pituitary–gonadal axis regulates puberty by the secretion of sex steroids to initiate and maintain the physical changes of puberty^28^. Previous studies reported that MC3R is involved in the regulation of puberty in both sexes, including the timing of the onset, linear growth rate and the accrual of lean mass^21,29,30^. A recent GWAS for human height reported a single nucleotide polymorphism (SNP) located in the upstream region of *MC3R* (distance 0.37 kb) that is associated with body height (rs6127698, effect allele = T, *β* = − 0.015, *p* = 3.96 × 10^−43^)^31^.

To further investigation of the impact of variants in *MC3R* in the etiology of obesity and SNS, we sequenced the *MC3R* in children or adolescents with severe obesity and in children with SNS and healthy lean individuals as control group.

## Results

### Identification of 11 variants in the coding region of *MC3R*

**Figure 1:**
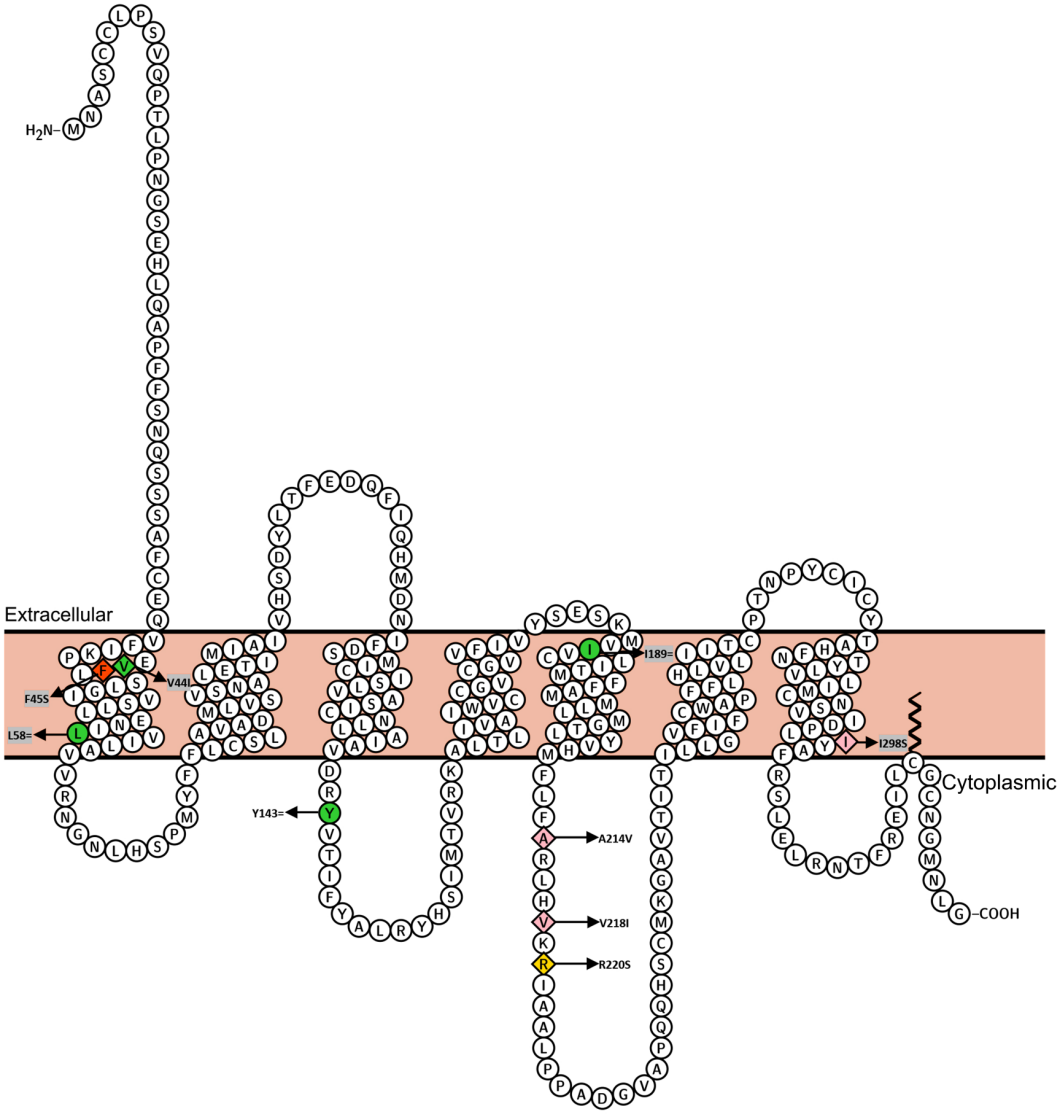
Identified variants at the *MC3R*. Synonymous variants are indicated with green circles; NSVs were depicted with rhombuses. ‘wildtype (WT)-like’ variant p.Val44Ile is green; partial loss-of-function (pLoF) variant p.Arg220Ser is yellow; complete loss-of-function (cLoF) variant p.Phe45Ser is red^21^; the variants with unknown function are colored in pink. The protein topological structure is generated according to the UniProt databank (UniProt ID: P41968)^32^.

We sequenced the *MC3R* coding region in study groups of 443 children or adolescents, comprising 185 with SNS, 258 with severe obesity (n = 258), and 192 healthy-lean adults as a control group. Eleven SNPs were identified (Fig. 1, see Supplementary material Table 1 online), including six non-synonymous variants (NSVs).

The frequent NSV p.Val44Ile was detected in all three groups with no significant differences in minor allele frequency (MAF) between them (MAF in SNS = 11.62%; MAF in obesity = 10.08%; MAF in healthy-lean = 9.11%). Three rare NSVs (MAF < 1%) (rs143321797 [p.Phe45Ser], rs61735259 [p.Arg220Ser], rs757322252 and rs121913556 [p.Ile298Ser]) were identified only in individuals with severe obesity. The novel NSV p.Ile298Ser, consisted of two adjacent nucleotide exchanges (rs757322252 [c.892 A > T], rs121913556 [c.893 T > C]) was detected once in a male obese patient. We preformed additional Sanger sequencing for *MC3R* in both mother and sister of the male heterozygous p.Ile298Ser carrier. Both adjacent SNPs were also identified in the mother, but not in the sister. Thus, the rare alleles (c.892T and c.893C) are most likely located on the same haplotype. Two rare NSVs (rs767076441 [p.Val218Ile], one novel NSV [p.Ala214Val]) were identified each once in the lean group. Two synonymous variants (SVs) (rs145062060 [c.172 C > T] and rs749736842 [c.174 G > A]) both have an effect on the 58th amino acid (leucine). The other two SVs (rs148382606 [p.Tyr143=] and rs41274722 [p.Ile189=]) were each identified only once in one healthy-lean individual (female) and one child (male) with SNS, respectively.

### The frequent NSV p.Val44Ile in SNS

Table 1 shows Hardy–Weinberg Equilibrium (HWE) calculations for different study groups. The frequent NSV p.Val44Ile significantly (*p* = 0.012) deviated from HWE in children and adolescents with SNS (independent patients). The observed number of heterozygous individuals was lower than expected and more individuals of both homozygous genotypes. The genotype distributions of p.Val44Ile were in HWE for the study groups of individuals with severe obesity or leanness.

**Table 1:** HWE calculation for p.Val44Ile in different study groups.

Study groups	Detected^a^			Expected^b^			Allele frequency^c^		HWE^d^
	11	12	22	11	12	22	1 (%)	2 (%)	
SNS	148	31	6	145.5	38	2.5	88.38	11.62	0.012
Obesity	208	48	2	208.6	46.7	2.6	89.9	10.1	0.670
Healthy-lean	158	33	1	158.6	31.8	1.6	90.9	9.1	0.604
1000G_CEU	83	15	1	82.7	15.5	0.7	91.4	8.6	0.729
1000G_EUR	429	71	3	428.9	71.1	3.0	92.4	7.7	0.973
gnomAD2_CEU	21,206	3878	181	21,202.9	3884.2	177.9	91.6	8.4	0.799
gnomAD2_EUR	53,679	10,165	487	53,674.2	10,174.6	482.2	91.3	8.7	0.810
gnomAD3_EUR	28,427	5304	269	28,409.0	5340.1	251.0	91.4	8.6	0.213

^a^Detected: The genotype distribution of p.Val44Ile in different study groups.

^b^Expected: expected genotype distribution of p.Val44Ile calculated with allele frequencies and sample sizes.

^c^Allele Frequency: the frequencies of allele 1 (p.Val44) and allele 2 (p.44Ile) in the study groups.

^d^HWE: calculated *p* values of a 1-df chi^2^-Test for HWE.

To investigate whether this SNP generally deviates from HWE in European populations, we extracted genotype distributions from individuals of European ancestry (North-western European [CEU], all European [EUR]) from two reference databases (1000 genomes Project [1000G], gnomAD [in two versions: V2.1.1, V3.1.2]). Genotypes of all other samples were in HWE (Table 1, Supplementary material Table 2 online).

Samples with variant patterns were confirmed with sequencing of the other strand, and sequencing was repeated once. As we analyzed all genotypes for p.Val44Ile in SNS twice and confirmed them, we had no indication to suspect that the deviation from HWE in the SNS study group was due to genotyping errors. We did not detect associations between genotypes or allele frequencies and SNS compared to the lean healthy control group (see Supplementary material Table 3 online).

For association analyses of the frequent NSV p.Val44Ile with BMI or BH-centiles, the study groups were divided into three genotypes (p.Val44/p.Val44, p.Val44/p.44Ile, p.44Ile/p.44Ile) and statistical test were performed (summarized in Supplementary material Table 4 online). The association of dosage effects of the allele p.44Ile on BMI or BH-centiles were analyzed with non-parametric Kruskal–Wallis test and Dunn’s multiple comparisons test for pairwise comparison (for two-tailed exact *p* values see supplementary material Table 5 online) in the separated study groups. A total of nine tests were performed, the corrected threshold for *p* values is 0.0055. Comparisons of BMI for the three genotypes in individuals with SNS (combined sexes) showed no differences after correction for multiple testing (uncorrected *p* = 0.0194). We observed a similar result for males with SNS (uncorrected *p* = 0.0396). Pairwise comparisons showed the homozygous p.44Ile carriers with SNS had nominally higher BMI than the individuals homozygous for the other allele before correction for multiple testing (uncorrected *p* = 0.0216). The increased BMI of homozygous p.44Ile male carriers compared to homozygotes for p.Val44 (uncorrected *p* = 0.0373) and heterozygotes for p.Val44Ile (uncorrected *p* = 0.0478) was considered exploratory and not significant after correction for multiple testing. The BMI and BH-centiles did not differ between genotype groups within patient groups with obesity or SNS and healthy lean individuals.

### Association analyses for detected rare variants

Association tests were conducted for the detected rare *MC3R* variants for both sexes combined and separately (refer to Supplementary material Table 6 online) and multiple testing was corrected using Bonferroni correction for 21 tests. We did not detect association between genotypes and traits.

### *In-silico* analyses for all detected variants

*In-silico* analyses were performed for all detected variants (Table 2). Generally, conservation was high for all detected NSVs (higher than 85%, detailed results showed in Supplementary material Table 7 online). Additional *in-silico* analyses ensued to quantify the scale of possible pathogenesis of mutated positions (detailed results showed in Supplementary material Table 8 online). Four missense variants (p.Phe45Ser, p.Ala214Val, p.Arg220Ser, and p.Ile298Ser) were predicted as pathogenic, including stability alteration in all *in-silico* tools. The novel variant p.Ala214Val was the only variant that may increase the protein stability.

**Table 2:** *In-silico* analyses for detected *MC3R* variants.

SNP-ID^a^	AA exchange^b^	Con_perc (%)^c^	Nuc_del (i/3)^d^	AA_del (j/2)^e^	P_stab (k/2)^f^
rs3827103	p.Val44Ile	100	1/3	0/2	dec (2/2)^i^
rs143321797	p.Phe45Ser	100	3/3	2/2	dec (2/2)
rs145062060	p.Leu58 =	100	1/3	NA^h^	
rs749736842	p.Leu58 =	76.92	1/3	NA	
rs148382606	p.Tyr143 =	84.62	2/3	NA	
rs41274722	p.Ile189 =	30.77	2/3	NA	
Novel^g^	p.Ala214Val	100	3/3	2/2	inc (2/2)^j^
rs767076441	p.Val218Ile	88.46	0/3	0/2	dec (2/2)
in rs61735259	p.Arg220Ser	100	3/3	2/2	dec (2/2)
rs757322252	p.Ile298Ser	100	3/3	2/2	dec (2/2)
rs121913556			3/3		

^a^SNP-ID: the dbSNP ID of detected variants.

^b^AA exchange: AA alteration of detected variants.

^c^Con_perc (%): the percentile of conserved positions in the analyzed 26 species.

^d^Nuc_del (i/3): the altered nucleotide was predicted as deleteriousness in i of three *in-silico* tools.

^e^AA_del (j/2): the alternative AA was evaluated as pathogenic in j of two tests.

^f^P_stab (k/2): the protein stability changing in k of 2 predictors.

^g^Novel: the novel variant that has not been identified in the previous studies.

^h^NA: not available data.

^i^dec (2/2): protein stability decreased in both estimated software.

^j^inc (2/2): protein stability increases in both software.

All detected variants with known dbSNP number were looked up in four GWAS datasets (see Supplementary material Table 9 online) for BMI^33^, body height^29^ and puberty time^29,30^. The minor allele A of frequent NSV (p.Val44Ile, rs3827103) was associated with later male and female puberty timing (male puberty: effect allele = A, *β* = 0.038, *p* = 3.77 × 10^−11^; female puberty: effect allele = A, *β* = 0.053, *p* = 8 × 10^−11^). For 35 of our patients with SNS (26 males and nine females) X-ray films on the hands of the participants showed that 31 of them had a bone age retardation of at least 1 year (23 males and eight females)^12^. Unfortunately the small number of available data does not allow for meaningful statistical analyses of the 35 individuals.

### Interacting network for *MC3R*

The interacting network of MC3R was generated by GeneMANIA^65^, which includes 20 genes shown in online Supplementary material Table 10. The genes were looked up in previous studies and the GWAS Catalog to explore their relevance to body weight, body stature, or puberty timing. Fourteen genes were demonstrated as BMI associated in either previous studies or genome-wide significant (*p* < 5 × 10^−8^) in GWAS datasets. Moreover, three genes (*MC4R*, *POMC*, and *MRAP2*) are known to be relevant for obesity^34^. Besides the impact of these genes on body weight, eleven and eight of the 20 interacting genes play roles in body height and puberty, respectively (showed in Supplementary material Table 10 online).

## Discussion

Recent studies have shown that MC3R has a non-redundant effect with the MC4R on body weight regulation and it is relevant to body height and puberty timing^21,26,27^. Our previous study on the *MC4R* mutations in SNS did not reveal rare *MC4R* variants leading to reduced MC4R function in short normal stature^35^. Consequently, we Sanger sequenced the coding region of *MC3R* in 185 and 258 children and adolescents with SNS or severe obesity, respectively, and 192 healthy lean individuals. Six NSVs and three SVs were identified, including two novel SNVs (one combined NSV [comprising of two altered nucleotides] p.Ile298Ser and p.Ala214Val). One female heterozygous p.Val44Ile carrier was also heterozygous for the *MC4R* variant p.Thr112Met. No other individuals were detected with variants in both genes^35^.

### The frequent NSV p.Val44Ile may have an effect on BMI in SNS

Deviation from HWE is typically considered a consequence of genotyping errors or population substructure^36^. A significant deviation (*p* = 0.012) was observed in the SNS group but not in the other study groups and reference databases (1000G project, gnomAD V2.1.1, gnomAD V3.1.2). We excluded the probability of genotyping errors with bidirectional Sanger sequencing and analyses by two expert scientists. All recruited patients were from unrelated families.

This pattern of deviation from HWE could potentially be caused by different population genetic mechanisms. In particular, population substructure occurs due to e.g. assortative mating in the parental generation, inbreeding (strong inbreeding is unlikely in the German population or population isolates (again, unlikely in our sample). Body height is a considerable factor in mate choice, and many studies showed that male stature is a key predictor of the mating preferences of females^37,38^. However, other studies stated that there is no association between stature and mating^39,40^. Due to the lack of detailed information on paternal phenotypes, we cannot elucidate if the deviation was led by assortative mating or inbreeding. Thus, the deviation of HWE for p.Val44Ile in SNS might imply a relevance for the allele p.44Ile in SNS.

We then estimated dosage effects of p.44Ile on body weight or body height through comparisons of BMI and BH-centiles among different genotypes of p.Val44Ile (homozygotes for p.Val44, heterozygotes for p.Val44Ile, homozygotes for p.44Ile) in each diagnostic group (children or adolescents with SNS or obesity, healthy lean individuals). The effect of three genotypes on BMI in children and adolescents with SNS was nominally different in the combined-sex group and in the male group. Besides, pairwise comparison showed nominally increased BMI in homozygous p.44Ile carriers with SNS, especially for male patients. In contrast, individuals who were heterozygous for p.Val44Ile had a similar BMI compared to individuals who are homozygotes for p.Val44. Although after Bonferroni correction for nine tests (significance *p* < 0.0055) none of the tests reached significance, the clear tendency and putative BMI increased effects of homozygotes for p.44Ile can be observed.

BMI is an indirect description parameter without distinguishing body composition. A previous study showed that a higher ratio of fat mass (FM) to lean body mass (LBM) could be observed in many syndromes related to short stature, such as Prader-Willi syndrome (PWS)^41^ and Silver-Russell syndrome (SRS)^42,43^. Lam et al. showed a negative correlation between p.Val44Ile and total LBM (*β* =  − 59, 95% CI = [− 98, − 19], *p* = 0.004; *p* values are determined by the non-infinitesimal mixed model association test from BOLT-LMM) and lean mass index (*β* =  − 0.013, 95% CI = [− 0.024, − 0.001], *p* = 0.036)^21^. In vivo studies showed that increased FB and decreased LBM in Mc3r dysfunctional mice^24,25^. Thus, we assume that the allele p.44Ile may reduce or disrupt the MC3R protein function and upregulate BMI through elevated FM and decreased LBM.

We did not observe a significant association between allele p.44Ile and BMI in individuals with obesity or leanness. No association between BH-centiles and p.Va44Ile genotypes was detected in any of the three study groups. Previous studies in humans reported that p.44Ile is associated with delayed age at puberty for both sexes^21,29,30^. Consequently, we assume that p.Val44Ile is relevant in body weight/height regulation and puberty onset.

### Four rare NSVs may trigger functional consequences

Delayed puberty timing, reduced linear growth rate and lean mass, as well as downregulated circulating levels of IGF1 in humans due to LoF mutations in *MC3R* were reported^21^. The missense variant p.Phe45Ser was demonstrated as a complete loss-of-function (cLoF) variant by measurement of cAMP generation ability in human embryonic kidney cell line (HEK294)^21^. It was found twice in our obese study group (heterozygous in a female and a male with obesity) and was detected in heterozygotes with severe obesity in previous studies^44^. The variant allele carriers had a delaying effect on female and male pubertal onset and reduced adult body height^21^.

Three other highly conserved NSVs (p.Ala214Val, p.Arg220Ser and p.Ile298Ser) were predicted as deleterious in all *in-silico* tools. The heterozygotes for p.Arg220Ser were only detected in the obese study group (twice in females, once in a male) and in a child with obesity in a previous study^44^. Previous studies in mice and in humans showed that the p.Arg220Ser leads to a partial loss-of-function (pLoF) with decreased cAMP activity and is associated with delayed puberty and reduced body height^21,29^. Although the combined missense variant p.Ile298Ser (rs757322252, rs121913556) was not examined in previous functional analysis, the mutated protein with the same amino acid alteration (rs121913556, T > G) was described as associated with susceptibility to obesity and causing cLoF and detected only in obese patients in previous studies^45,46^.

The novel NSV p.Ala214Val was detected once in a female lean individual. It is highly conserved (100% conserved in 26 species) and may increase the stability of the mutated protein. The NSV p.Val218Ile with low conservation was detected once in a healthy female lean individual. This variant was predicted as polymorphism in five *in-silico* tools. Nevertheless, there is no evidence to show the putative functional consequences caused by this variant.

### Dysfunction of MC3R may lead to decreased body height, obesity and delayed puberty development

The initial GWAS lookup for all detected variants denoted that the frequent non-synonymous variant p.Val44Ile is genome-wide significantly associated with female and male puberty timing^29,30^. Two *MC3R* NSVs (p.Thr6Lys and p.Val81Ile), classified as V Class (mutants with unknown defects that behave like WT) are the most extensively studied variants^19,47–50^. There were some controversies regarding functions of these two NSVs. A double-mutant mice model carrying the two NSVs reported greater weight and higher ratio of fat mass, therefore provided evidence for the contribution of *MC3R* common variants in body weight and composition^51^. We detected three LoF NSVs (p.Phe45Ser, p.Arg220Ser and p.Ile298Ser) only in patients with obesity which had been reported to be associated with decreased body height or obesity or delayed puberty^21,45,46^. Therefore, *in-vivo* studies of these mutations will be a crucial step in describing effects of *MC3R* LoF variants on phenotypes.

Leptin and insulin are stimulators of pro-opiomelanocortin (POMC) neurons and neuropeptide Y (NPY)-agouti-related protein (AgRP) neurons which play a crucial role in energy balance controlling and hormone homeostasis maintenance^13,52^. The second messenger α-MSH from POMC neurons in arcuate nucleus (ARC) stimulates melanocortin systems (MC3R in anteroventral periventricular nucleus [AVPV] and MC4R in paraventricular nucleus [PVN]) and several neurons which modulate the secretion of growth hormone^21,53^. Fourteen, eleven and eight of the 20 genes interacting with *MC3R* had previously been demonstrated to be relevant for body weight, body height and puberty timing, respectively. Thus, MC3R may be involved in the regulation of body weight/height and puberty onset.

## Conclusion

Six NSVs and three SVs were detected in *MC3R* by Sanger sequencing in study groups comprising children or adolescents with SNS or severe obesity and healthy lean controls. The frequent NSV p.Val44Ile significantly deviated from HWE in SNS. We observed a putative effect of allele p.44Ile on increased BMI in individuals with SNS. However, the statistical test was non-significant after Bonferroni correction. We assume that the allele p.44Ile may have an effect on increased BMI (by increased FM and decreased LBM), decreased body height and delayed puberty in both sexes. Besides, three LoF NSVs were detected only in our obese patients and were reported to be involved in puberty onset and regulation of body weight and height. The novel missense variant p.Ala214Val may have increased protein stability. To sum up, MC3R may have a critical impact on body weight, body height, and puberty timing.

## Method

### Study group

We Sanger sequenced the coding region of *MC3R* in 631 German individuals including 185 children or adolescents with diagnosed SNS, 258 children and adolescents (younger than 25 years old) with (severe) obesity (87.6% of them were extremely obese with BMI-centiles ≥ 97th percentile^7^), and 192 healthy-lean individuals (BMI-centiles ≤ 15th percentile).

Body weight and height were measured using calibrated hospital scales and stadiometers. Participants were weighed in underwear without shoes. The ascertainment strategy of SNS was previously described in detail^35^. BMI was calculated by dividing weight by the square of height (kg/m^2^). Individual BMI-values were transformed into BMI-SDS and BMI-centiles using the method suggested by Cole^54^. The method was adapted for the calculation of BMI-SDS by Kromeyer-Hausschild et al.^55^. For participants younger than 19 years we used German reference data for children (Kromeyer-Hausschild et al.^55^), for adult participants the reference data by Hemmelmann et al.^56^ were used. The BMI-SDS approximates the deviation of an individual BMI from the median of the reference group expressed in units of the standard deviation. BMI-centiles, transformed from BMI-SDS, were applied to diagnose patients with obesity and identify the lean individuals (detailed data not shown). Using the same method^54^ and reference data for children and adolescence^55^, we also transformed individual body heights (BH) into BH-centiles to investigate potential age dependent effects of starvation induced stunting on body height. For adult individuals, the BH-centiles were not applicable.

The phenotypes of the study groups are shown in Supplementary material Table 11 online. Written informed consent was given by all participants and in case of minors also by their parents. The study was approved by the Ethics Committees of the Universities of Gießen, Marburg, Essen and was performed in accordance with the Declaration of Helsinki.

### Sanger sequencing

The *MC3R* gene (one coding exon) is located on chr20: 56 248 732–56 249 815 (GRCh38/hg38). The genomic sequences of the *MC3R* gene were extracted from the Ensembl Database (http://www.ensembl.org/index.html). The cDNA and protein sequence of the transcript variant 1 of the *MC3R* gene (MC3R-201, ENST00000243911.2) was used. Primer pairs were designed using the online software PRIMER3 (https://primer3.ut.ee/). Primers were analyzed using BLAST (https://blast.ncbi.nlm.nih.gov/Blast.cgi) and *in-silico* PCR (https://genome.ucsc.edu/cgi-bin/hgPcr) of the UCSC Genome Browser to verify the designed primer’s specificity (primer list is shown in Supplementary material Table 12 online). Polymerase chain reaction (PCR) amplified DNA samples were sequenced by Microsynth Seqlab GmbH (Göttingen, Germany). All sequences were analyzed using the SeqMan Pro software by DNAStar, Inc. (version: 10.1.0) and evaluated by two experienced scientists. Samples with variant patterns were confirmed with bidirectional sequencing. Hardy–Weinberg Equilibrium (HWE) was analyzed for all variants using a 1-degree of freedom chi^2^-test via Microsoft Excel. The detected variants were plotted in the amino acid sequence of MC3R by Protter (http://wlab.ethz.ch/protter/start/)^57^.

### Association analyses

Association analyses were performed for the identified variants via Fisher’s exact test (https://www.socscistatistics.com/tests/fisher/default2.aspx). If the variants were detected in obesity or patients with SNS and as well as in the lean control groups, then the variants were compared to the frequency of the alternative allele in the lean group. The genotype data was extracted from gnomAD V3.1.2 (European non-Finnish) for variants that were not detected in the lean group. To access the impact of frequent NSV dosage, which was detected in all three of our study groups (children and adolescents with SNS or obesity, healthy lean individuals), on the BMI and BH-centiles, statistical analyses were performed with Kruskal–Wallis tests and Dunn’s multiple comparisons tests using GraphPad Prism software (version 9.5.0). The statistical test was further performed for both sexes separately when at least two individuals for each genotype were counted. All reported *p* values are two tailed and the significance threshold was corrected with Bonferroni correction.

### *In-silico* analyses

#### Conservation analysis

The conservation analysis of human *MC3R* cDNA and protein sequences were compared to 25 other species (ten primates, five rodents and related species, five laurasiatherian, and five sauropsids) with the software MegAlign by DNAStar, Inc. (version 10.1.0) using the cluster W method.

#### *In-silico* functional analyses on detected variants in *MC3R*

All detected variants in *MC3R* were analyzed with eight *in-silico* tools. The predicted deleteriousness of single nucleotide exchanges were evaluated in MutationTaster (https://www.mutationtaster.org/)^58^, Combined Annotation dependent depletion (CADD, https://cadd.gs.washington.edu/snv)^59^ and PredicSNP2 (https://loschmidt.chemi.muni.cz/predictsnp2/)^60^. Two web-based software PolyPhen2.0 (http://genetics.bwh.harvard.edu/pph2/)^61^ and PROVEAN (http://provean.jcvi.org/index.php)^62^ were utilized to calculate pathogenic potentials of the altered amino acids. Then the stability of mutated protein with the amino acid sequences were analyzed via I-Mutant2.0 (http://gpcr2.biocomp.unibo.it/~emidio/I-Mutant2.0/I-Mutant2.0_Details.html)^63^ and iStable (http://predictor.nchu.edu.tw/istable/indexSeq.php)^64^. Predicted interacting genes were also analyzed and a putative network of MC3R was generated in GeneMANIA (https://genemania.org)^65^.

#### GWAS look up

The detected variants with known dbSNP-ID were looked up in four GWAS summary statistic datasets (Supplementary material Table 13 online). The associating genes of *MC3R* were looked up in the GWAS catalog (https://ebi.ac.uk/gwas/)^66^.

### Ethical approval and informed consent

Written informed consent was given by all participants and in case of minors by their parents. The study was approved by the Ethics committees of the Universities of Gießen, Essen, Marburg, and was performed in accordance with the Declaration of Helsinki.

## Supplementary Information

Supplementary Tables.

## Data Availability

The datasets used and/or analysed during the current study available from the corresponding author on reasonable request. The variants detected in this study have been submitted to the NCBI Variation Submission Portal (dbSNP, https://www.ncbi.nlm.nih.gov/SNP/snp_viewTable.cgi?handle=ESSENKJP). **Database** Pulit SL, Stoneman C, Morris AP, Wood AR, Glastonbury CA, Tyrrell J et al. Meta-analysis of genome-wide association studies for body fat distribution in 694 649 individuals of European ancestry. Human Molecular Genetics. 2018;28(1):166–74. https://doi.org/10.1093/hmg/ddy327. https://zenodo.org/record/1251813#.Yoy_pxNBw-R Hollis B, Day FR, Busch AS, Thompson DJ, Soares ALG, Timmers PR et al. Genomic analysis of male puberty timing highlights shared genetic basis with hair colour and lifespan. Nature communications. 2020;11(1):1–10. http://ftp.ebi.ac.uk/pub/databases/gwas/summary_statistics/GCST90012001-GCST90013000/GCST90012088/ Loh PR, Kichaev G, Gazal S, Schoech AP, Price AL. Mixed-model association for biobank-scale datasets. Nat Genet. 2018;50(7):906–8. https://doi.org/10.1038/s41588-018-0144-6. https://alkesgroup.broadinstitute.org/UKBB/ **Websites:** Archive Ensembl Database: http://www.ensembl.org/index.html; PRIMER3: https://primer3.ut.ee/; BLAST: https://blast.ncbi.nlm.nih.gov/Blast.cgi;*in-silico* PCR: https://genome.ucsc.edu/cgi-bin/hgPcr; Protter: http://wlab.ethz.ch/protter/start/; MutationTaster: https://www.mutationtaster.org/; CADD: https://cadd.gs.washington.edu/snv; PredicSNP2: https://loschmidt.chemi.muni.cz/predictsnp2/; PolyPhen2.0: http://genetics.bwh.harvard.edu/pph2/; PROVEAN: http://provean.jcvi.org/index.php; I-Mutant2.0: http://gpcr2.biocomp.unibo.it/~emidio/I-Mutant2.0/I-Mutant2.0_Details.html;iStable: http://predictor.nchu.edu.tw/istable/indexSeq.php; GeneMANIA: https://genemania.org; GWAS Catalog: https://www.ebi.ac.uk/gwas/ **Softwares:** SeqMan Pro (DNAStar, Inc., version: 10.1.0), GraphPad Prism 9.5.0.
